# Antibiotic resistance of *Escherichia coli* isolated from uncomplicated UTI in general practice patients over a 10-year period

**DOI:** 10.1007/s10096-019-03655-3

**Published:** 2019-08-22

**Authors:** A. A. van Driel, D. W. Notermans, A. Meima, M. Mulder, G. A. Donker, E. E. Stobberingh, A. Verbon

**Affiliations:** 1 Department of Medical Microbiology & Infectious Diseases, Erasmus University Medical Center, ‘s-Gravendijkwal 230, 3015 CE Rotterdam, The Netherlands; 2 National Institute for Public Health and the Environment (RIVM), Antonie van Leeuwenhoeklaan 9, 3721 MA Bilthoven, The Netherlands; 3 Department Infectious Disease Control, Municipal Public Health Service Rotterdam-Rijnmond (GGD Rotterdam), Rotterdam, The Netherlands; 4 Department of Epidemiology, Erasmus Medical Center, ‘s-Gravendijkwal 230, 3015 CE Rotterdam, The Netherlands; 5 Nivel Primary Care Database, Sentinel Practices, The Netherlands Institute for Health Services Research, Utrecht, The Netherlands; 6 Department of Medical Microbiology, Maastricht University Medical Centre, Maastricht, The Netherlands

**Keywords:** Antibiotic resistance, Uncomplicated urine tract infections, Community-acquired, ESBLs

## Abstract

Recommendations of first choice antibiotic therapy need to be based on actual antibiotic susceptibility data. We determined the antibiotic susceptibility of *E. coli* in uncomplicated UTI among women and compared the results with 2004 and 2009. In 30 sentinel general practitioner practices of Nivel Primary Care database, urine samples were collected from women with symptoms of uncomplicated UTI. Patient characteristics, *E. coli* susceptibility, and ESBL production were analyzed. Six hundred eighty-nine urine samples were collected; *E. coli* was the most isolated uropathogen (83%). Antibiotic susceptibility was stable over time except for ciprofloxacin (96% in 2004, 97% in 2009, and 94% in 2014; *P* < 0.05). The susceptibility to co-amoxiclav was 88%, 87%, and 92% in 2004, 2009, and 2014, respectively. The prevalence of ESBL-producing *E. coli* increased from 0.1% in 2004 to 2.2% in 2014 (*P* < 0.05). Regional differences in antibiotic susceptibility for co-trimoxazole were found being the highest in the west (88%) and the lowest in the north (72%, *P* = 0.021). Ciprofloxacin susceptibility was related to antibiotic use in the past 3 months (97% no use versus 90% use, *P* = 0.002) and age > 70 years (*P* = 0.005). In 2014, prescription of fosfomycin increased compared to 2009 (14.3% versus 5.6%) at the expense of co-amoxiclav, co-trimoxazole, and fluoroquinolones (*P* < 0.05). The susceptibility percentages to most antimicrobial agents tested were stable over 10 years’ period although the prevalence of *E. coli* and ESBLs significantly increased. Performance of a survey with regular intervals is warranted.

## Introduction

Most antibiotics in the Netherlands prescribed by general practitioners (GPs) are for uncomplicated acute urinary tract infections (UTI) in women [1, 2]. *Escherichia coli* (*E. coli*) is the most frequently isolated microorganism, in up to 80% of UTIs [3, 4]. Different studies report increased resistance of *E. coli* in UTI, especially to beta-lactam antibiotics and fluoroquinolones alone or in combination [5, 6]. Although antibiotic resistance in the Netherlands is one of the lowest worldwide, the prevalence of resistant microorganisms has been increasing over time. Several studies describe an increasing prevalence of extended spectrum β-lactamase (ESBL)-producing Gram-negative rods and other highly resistant microorganism (HRMO) in the general population [7–9]. In addition, a high prevalence of resistant microorganisms has been reported in specific populations such as travelers returning from South East Asia [10], immigrants and refugees [11, 12], and patients with hospital-replaced home treatment [13], as well as in livestock and agriculture [14–17]. Therefore, to make an optimal empiric choice, it is important to determine with regular time intervals the antibiotic susceptibility in populations commonly treated such as women with UTI.

For women with UTI, treatment guidelines are published by the Dutch College of General Practitioners (NHG). The choice of antimicrobial agents in the guidelines is based on national antimicrobial resistance (AMR) data published in Nethmap [8]. These data are derived from microbiological laboratories sending data to the National Institute for Public Health and the Environment. Yearly, this institute in close cooperation with the SWAB (Dutch Working group on Antibiotic policy) reports national data on antibiotic consumption and resistance in humans and animals in Nethmap and MARAN, respectively [8]; based on the NHG guideline, GPs should only submit urine for culture after failure of first-line therapy [18]. However, antimicrobial resistance of uropathogens isolated after therapy failure is not representative for resistance rates in patients who were successfully treated. Since NHG guidelines for treatment of UTIs should be based on the latter population, we determined the AMR among women with complaints of an acute uncomplicated UTI visiting their GPs. Additionally, to determine trends in susceptibility, AMR data of 2014 were compared with the data collected in a similar population in 2004 and 2009 [19, 20].

## Patients and methods

### Patients

From January 2014 to January 2015, all women of 11 years and older with symptoms of dysuria, strangury, or urinary frequency visiting 30 sentinel GP practices participating in Nivel Primary Care Database were included [21]. This GP network is nationally representative by regional distribution, population density, practice features, GP, and patient characteristics [21]. Exclusion criteria were symptoms of complicated UTI, fever > 38 °C, pregnancy, diabetes mellitus, urological or nephrological problems, or immunodeficiency of any kind, as were also antibiotic use and an episode of recurrent UTI in the past 4 weeks.

To avoid bias concerning age, region, or medical history of the patient, a maximum of 25 samples from any GP practice was included during the study period.

Antibiotic prescription and UTI treatment history in the past 3 months were recorded. A urine dipslide (Uriline, 56508; BioMérieux, Plainview, NY USA) was inoculated according to the manufacturer’s instructions and sent for analysis to the Centre for Infectious Diseases Research, Diagnostics and laboratory Surveillance (IDS) of the National Institute for Public health and the Environment (RIVM) in the Netherlands.

### Ethics approval and consent to participate

For patient-derived data from the NIVEL-PCD, ethics approval via an METC was not needed, as explained in the “Privacy statement NIVEL PCD”: Dutch law allows the use of electronic health records for research purposes under certain conditions. According to this legislation, neither obtaining informed consent from patients nor approval of a medical ethics committee is obligatory for this type of observational studies containing no directly identifiable data (Dutch Civil Law, Article 7:458; http:// www.dutchcivillaw.com/civilcodebook077.htm). This study has been approved by the applicable governance bodies of NIVEL Primary Care Database under nr. NZR00316.012.

### Identification of the uropathogens and antibiotic susceptibility testing

Bacterial growth on the dipslide was considered positive at ≥ 10^5^ cfu/mL. Growth of two or more bacterial species was considered as contaminated and excluded. For identification of the uropathogens, MALDI-TOF MS (MALDI Biotyper 3.0 van Bruker Daltonik GmbH, Bremen, Germany) was used. The isolated microorganisms were stored at − 20 °C in peptone/glycerol (30% w/v).

The antibiotic susceptibility of the isolates was performed by disk diffusion according to the EUCAST criteria version 4.0 valid from Jan 1, 2014 as were the breakpoints of susceptibility. The following antibiotics were tested: amoxicillin (10 μg), co-amoxiclav (20/10 μg), trimethoprim (5 μg), co-trimoxazole (1.25/23.75 μg), ciprofloxacin (5 μg) nitrofurantoin (100 μg), and fosfomycin (200 μg). *Escherichia coli* ATCC 35218 and ATCC 25922 were used as control strains; breakpoints of susceptibility were according to the EUCAST guidelines (http://www.eucast.org/clinical_breakpoints/). The susceptibility of fosfomycin was tested using the CLSI guidelines [22]. For detection of ESBL-producing bacteria, putative colonies were streaked on ChromID ESBL agar (Biomerieux) and confirmation of ESBL production was performed according to the guideline of the Dutch Society of Medical Microbiology (NVMM) [23].

### Comparison with the 2004 and 2009 data

The GP network, patient population, and methods were comparable to those described in the 2004 and 2009 studies, as were the criteria for inclusion and exclusion of patients. The microbiological analysis was performed in another laboratory using the same methods as described for the 2004 and 2009 studies [19, 20, 22, 23]. The difference with the studies of 2004 and 2009 was the cutoff to define a UTI using a colony count of 10^5^ cfu/mL in the present study instead of 10^3^ cfu/mL in the previous ones. The EUCAST version used was version 1.0 tentative for the period 2002–2009 and version 1.2 valid from December 2010 in the previous studies and EUCAST version 4.0 valid from January 2014 in the present one (http://www.eucast.org/clinical_breakpoints/).

### Regional differences

To analyze regional differences, we used the regional distribution as used by Nivel [21]: North (provinces Groningen, Friesland, and Drenthe), East (Overijssel, Gelderland, and Flevoland), West (Utrecht, North-Holland, and South-Holland), and South (Zeeland, North-Brabant, and Limburg) [21].

### Statistics

For the statistical analysis, SPSS 23.0 was used. The *X*^2^ test was used to test the significance of associations within the data.

## Results

### Baseline characteristics

During the study period, 689 urine samples were collected, of which 535 (78%) were considered positive, 117 (17%) negative, and 37 (5%) were excluded because two or more bacteria species were isolated. The median age of the total group of all women was 57 years (range 11–101 years). Antibiotic use in the past 3 months was the lowest in the age group 11–20 years (17%) and increased to 46% in the age group of 70 years and older. *E. coli* was the most frequently detected uropathogen (83%), followed by *Klebsiella pneumonia* (5%) and *Klebsiella oxytoca* (1.5%).

### Antibiotic susceptibility of *E. coli*

Table 1 shows the susceptibility of *E. coli* in relation to three determinants, i.e., antibiotic use in the past 3 months, age, and region. Overall, the susceptibility ranges from 66% for amoxicillin to 94% for ciprofloxacin and nearly 100% for both nitrofurantoin and fosfomycin. For the various antibiotics, only three statistically significant associations were found between susceptibility and any of the three determinants. For co-trimoxazole, the association with region was significant: highest susceptibility in the west (88%) and lowest in the north (72%, *P* = 0.021). For ciprofloxacin, the associations with both previous antibiotic use and age were significant: less susceptibility when previous antibiotic use (90% vs 97%) (*P* = 0.002) and age > 71 (88%) (*P* = 0.005). The association with region could not be tested for ciprofloxacin (violation of chi-square test assumptions). Only 26, 3, and 2 isolates were resistant to ciprofloxacin, nitrofurantoin, and fosfomycin, respectively.

**Table 1:** Antibiotic susceptibility of *E. coli* according to previous antibiotic use, age, and region

	Amoxicillin	Co-amoxiclav	Trimethoprim	Co-trimoxazole*	Ciprofloxacin**	Nitrofurantoin***	Fosfomycin***
Antibiotic use in the last 3 months susceptibility in number and (percentage)							
Yes (*N* = 144)	87 (60%)	131 (91%)	108 (75%)	111 (77%)	129 (90%)	142 (99%)	142 (99%)
No (*N* = 282)	196 (70%)	261 (93%)	228 (81%)	235 (83%)	273 (97%)	281 (100%)	282 (100%)
Total (*N* = 426)^i^	283 (66%)	392 (92%)	336 (79%)	346 (81%)	402 (94%)	423 (99%)	424 (100%)
Age							
11–20 (*N* = 28)	19 (68%)	25 (89%)	22 (79%)	23 (82%)	26 (93%)	28 (100%)	28 (100%)
21–50 (*N* = 136)	88 (65%)	129 (95%)	107 (79%)	112 (82%)	131 (96%)	136 (100%)	136 (100%)
51–70 (*N* = 155)	107 (69%)	143 (92%)	125 (81%)	127 (82%)	151 (97%)	153 (99%)	154 (99%)
> 70 (*N* = 126)	79 (63%)	110 (87%)	99 (79%)	101 (80%)	111 (88%)	125 (99%)	125 (99%)
Total (*N* = 445)	293 (66%)	407 (91%)	353 (79%)	363 (82%)	419 (94%)	442 (99%)	443 (100%)
Region							
North (*N* = 82)	53 (65%)	73 (89%)	59 (72%)	59 (72%)	72 (88%)	81 (99%)	82 (100%)
East (*N* = 71)	50 (70%)	64 (90%)	55 (77%)	56 (79%)	64 (90%)	70 (99%)	71 (100%)
West (*N* = 184)	125 (68%)	169 (92%)	153 (83%)	161 (88%)	178 (97%)	184 (100%)	182 (99%)
South (*N* = 108)	65 (60%)	101 (94%)	86 (80%)	87 (81%)	105 (97%)	107 (99%)	108 (100%)
Total (*N* = 445)	293 (66%)	407 (91%)	353 (79%)	363 (82%)	419 (94%)	442 (99%)	443 (100%)

^i^In the case of antibiotic use in the last 3 months, 19 cases were unknown

*Association between susceptibility and region (*P* = 0.021)

**Associations between susceptibility and both previous antibiotic use (*P* = 0.002) and age (*P* = 0.005). For region, testing is inappropriate due to small numbers

***Testing is inappropriate due to small numbers

Of the 10 ESBL (2.2%)-positive isolates, nine were in the age group 21 to 70 years, eight of them were from the Eastern region.

### Comparison with the survey of 2004 and 2009

The prevalence of *E. coli* isolates increased from 64% (1013/1583) in 2004 to 72% (350/487) in 2009 [23], and to 83% (445/535) in 2014 (2004–2014, *P* < 0.05). No significant differences in prevalence were observed for the other isolated uropathogens (data not shown).

### Antibiotic prescriptions in 2009 and 2014

There was a significant difference in empiric antibiotic prescriptions between 2009 and 2014 (*P < 0.05*). The empirical prescribing of nitrofurantoin and fosfomycin increased, whereas the prescribing of the other antibiotics decreased (Table 2). The antibiotic use between 1999 and 2014 fluctuated between 10 and 11 DDD/1000 inhabitant-days [24].

**Table 2:** Number (%) of empiric antibiotic prescriptions in the 2009 and 2014 surveys

	2009	2014
	*N* = 719	*N* = 419
Amoxicillin, *N* (%)	13 (1.8)	2 (0.5)
Co-amoxiclav, *N* (%)	54 (7.5)	18 (4.3)
Trimethoprim, *N* (%)	38 (5.3)	14 (3.3)
Co-trimoxazole, *N* (%)	27 (3.8)	3 (0.7)
Fluoroquinolones, *N* (%)	75 (10.4)	29 (6.9)
Nitrofurantoin, *N* (%)	472 (65.6)	293 (69.9)
Fosfomycin, *N* (%)	40 (5.6)	60 (14.3)

Fluoroquinolones; ciprofloxacin, norfloxacin

Azithromycin (*n* = 3) prescription excluded

Unknown (*n* = 2) in 2014

(*X*_2_ test; *P* < 0.05)

In 2014, empirical antibiotic treatment was prescribed to 424 of the 689 patients (61.5%), and unknown in 70 (10.2%) patients. The most frequently prescribed antibiotic was nitrofurantoin (*n* = 293, 69%), followed by fosfomycin (*n* = 60, 14%) and ciprofloxacin (*n* = 25, 6%); the last two antibiotics were especially used in the elderly (> 51 years), 38% and 16%, respectively.

### AMR from 2004 to 2014

Table 3 compares antibiotic susceptibilities of *E. coli* over time. The susceptibility to co-amoxiclav was in the 2004 study 88% and in 2009 87% compared to 92% in 2014. The susceptibility for ciprofloxacin decreased significantly from 97% in 2009 to 94% in 2014 (*X*^2^ test; *P* < 0.05), but the decrease from 2004 (96%) to 2014 was not significant (*X*^2^ test; *P* = 0.10). No other differences in susceptibility to the other agents over time were found.

**Table 3:** Total *E. coli* susceptibility (%) in 2004 (Nys), 2009 (den Heijer), and 2014

	2004	2009	2014
	*N* = 1378	*N* = 489	*N* = 445
Amoxicillin	923 (67)	323 (66)	293 (66)
Co-amoxiclav	1213 (88)	425 (87)	407 (92)
Trimethoprim	1061 (77)	396 (81)	353 (79)
Co-trimoxazole	1102 (80)	411 (84)	363 (82)
Ciprofloxacin*	1323 (96)	474 (97)	419 (94)
Nitrofurantoin**	1364 (99)	489 (100)	442 (99)
Fosfomycin**	1364 (99)	489 (100)	443 (99)

* Indicates a significant decreased antibiotic susceptibility between 2009 and 2014 for ciprofloxacin (*X*^2^ test; *P* < 0.05)

**Numbers too low for chi-squared test

The prevalence of ESBL-producing *E. coli* increased over time from 0.1% (1/1378) in 2004, to 1% (5/489) in 2009 and to 2.2% (10/445) in 2014. The numbers involved do not allow for testing the significance of this increase.

## Discussion

In this study, we demonstrate that AMR did not increase over the 10-year study period except the resistance to ciprofloxacin and the prevalence of ESBL-producing *E. coli*. These results are remarkable in this time of worldwide concern on the increase in prevalence of antibiotic resistance [5, 6]. We also showed that the prevalence of *E. coli* isolated from urine significantly increased in this time period and that fosfomycin was more frequently prescribed at the expense of co-amoxiclav, co-trimoxazole, and fluoroquinolones.

In contrast to the global tendency of increasing AMR [25], we showed only small changes in antibiotic susceptibility over a 10-year time period. Both in Europe and on other continents, AMR has been increasing in bacteria causing UTIs [5, 26–28]. The Netherlands has always reported relatively low AMR rates and prudent antibiotic use [29]. Antibiotic consumption has been directly related to AMR and the low antibiotic use in the Netherlands may be an explanation for the stable AMR [30–32]. It should be noted that the cultures were taken from community-dwelling females with uncomplicated UTIs and a presumed low antibiotic use and pressure.

The susceptibility of *E. coli* to co-amoxiclav increased from 88% and 87% in the 2004 and 2009 studies to 92% in the present one. In the last study, the EUCAST criteria version 4.0 valid from January 2014 was used, whereas the two other studies used the version 1.2. The main difference is the breakpoint for susceptibility for co-amoxyclav being > 16 mm for all infections according to the version 1.2 and > 15 mm for UTI only is mentioned in the 4.0 version. The version 1.2 does not mention the criterium “UTI only”; in the 4.0 version, the breakpoint for all infections is > 18 mm. Due to the different breakpoints used and depending on the type of infections, it is difficult to assess whether the increase in susceptibility is a real increase of just a virtual one. In other European countries, a decrease of susceptibility has been described [26]. In Ireland, the susceptibility of *E. coli* to co-amoxiclav decreased from 90% in 2005 to 80% in 2014 in community-dwelling patients with UTI [27]. Also in Germany, co-amoxiclav susceptibility decreased from 92% in 2014 to 75% in 2017 [28].

The decreased susceptibility of *E. coli* to ciprofloxacin was in line with other studies [26–28]. In the EC0-SENS study, a significant increase of resistance to ciprofloxacin from 2000 to 2014 was observed in different countries: in Germany from 2.2 to 20.2%, in Spain from 14.7 to 30.8%, in Sweden from 0.0 to 7.3%, and in the UK from 0.6 to 15.3% [26]. In Ireland, resistance to ciprofloxacin increased from 5% in 2005 to 11.4% in 2014 [27]. An average increase in resistance per year of 0.40% and 0.26% was found both in community-dwelling and hospitalized patients, respectively [28]. According to the Dutch NHG guidelines, ciprofloxacin is a last resort drug and we showed that prescription rates decreased from 14% in 2004, 10% in 2009, to 5.9% in 2014 [18]. However, only a small effect on susceptibility percentages could be demonstrated; the *E. coli* susceptibility decreased from 96% in 2004 to 94% in 2014 (Table 3).

The rise in prevalence over time of *E. coli* was a remarkable finding. Although the microbiological analysis of our last survey was performed in another laboratory, the methods were comparable. The 2009 survey showed already a significant increase in prevalence of *E. coli* compared to 2004 [20]. The prevalence of *E. coli* in UTI ranged from 76.7 to 84% in different studies [27, 32, 33]. Cullen et al. also described an increase of *E. coli* in an 11-year period of uncomplicated UTI in community patients including fluctuation over time [33]. Different explanations were described in the literature, such as rise in global temperature, seasonal peaks, and increasing age of women with symptoms of UTI [34–36]. In this study, we included patients during 6 months and did not observe influences of these seasons or age for the percentage of *E. coli* isolates.

In Belgium, Heytens et al. found in a 20-year survey from 1995 to 2015 a higher percentage of *E. coli* in uncomplicated UTI in post-menopausal women > 55 years of age compared to the age group of 18–54 years, 89.9% versus 78.4%, respectively [36]. Although the mean age in our study was 53.5 years and 57% was 51 years or older, the prevalence of *E. coli* in the age group 51 years and older was comparable to the younger age group (82% versus 84%, respectively).

Other uropathogens such as *K. pneumonia* and *K. oxytoca* were isolated less frequently. Risk factors for uropathogens other than *E. coli*, like recurrent UTI, pregnancy, intensive care hospitalization, presence of catheters, gender, age, and diabetes mellitus, were exclusion criteria in our study except age and gender [36, 37].

The regional differences in susceptibility of *E. coli* (Table 1) were in contrast to the regional antibiotic prescription of these agents (Table 2)*.* The Northern and Eastern regions are both adjacent to Germany, where, as shown in the updated ECO-SENS study, resistance to ciprofloxacin increased from 2.2 to 20.2%, and trimethoprim from 22.5 to 36.8% from 2000 to 2014 [26]. Social and economic traffic between the regions and Germany is common [38] and geographical boundaries are not recognized by microorganisms [39]. However, no data is available of *E. coli* susceptibility in uncomplicated UTI in the community in the cross-border region. We found only limited regional data on antibiotic resistance in Belgium or in Germany. The only available numbers we found in Belgium were described in the relatively small study of De Backer (2008) in the region Gent with 187 patients [40]. He described in his study from 1996 to 2006 a stable prevalence of antibiotic susceptibility in urinary isolates, i.e., a susceptibility of 83.3 to 86% for co-trimoxazole, versus 86% in the South and 83% in the Western part of the Netherlands (Den Heijer 2009). Van den Donk et al. compared the prevalence of resistance in the cross-border region between Belgium, Germany, and the Netherlands among general practitioners, nursing homes, and ICU isolates and found a significant difference in prevalence of co-amoxiclav and ciprofloxacin resistance among all *E. coli* isolated between the Netherlands and Belgium, i.e., 27% versus 21%, and 23% and 16%, respectively. No significant differences with Germany were reported [41].

We found a regional difference in prevalence of ESBL-positive isolates, where 8 out of 10 isolates all in the age group of over 51 were from the Eastern region, close to the border of Germany. The ECO-SENS study in 2014 reported an ESBL incidence of 10.5% [26]. Van den Donk et al. (2012) showed a cross-border spread of *E. coli* ST131 and CTX-M type ESBLs and a similar prevalence of resistant *E. coli* in the Euregio Meuse Rhine [42]. A recent study found the same ESBL CTX-M15 genotype in cross-border hospitals in the Northern part of the Netherlands and in Germany whereas in community isolates from the Netherlands, CTXM1 was found. The results of this small study suggested cross-border spread in clinical isolates. As no data was available from German community strains, no conclusion could be drawn concerning possible cross-border spread in the community [43].

The prevalence of ESBL-positive *E. coli* isolates increased from 0.1% in 2004 to 1.0% in 2009 and 2.2% in 2014. Several reports also mentioned an increase in ESBL-producing *E. coli* in community-acquired urinary tract infections. In Germany, an increase was found, from 1.2 to 4.8% in 8 years’ time [26], and more than 12% in a 10-year period in Ireland [27]. Of the different risk factors described, such as recurrent UTI in the past year, (recent) use of β lactam antibiotics or fluoroquinolones, and traveling to Asia, Middle East, and Africa [10, 44], we only had information for recurrent UTI.

The increase in nitrofurantoin and fosfomycin as empirical prescription by the participating GPs was in line with the first and second choice of antibiotic treatment of uncomplicated UTI according to the NHG guidelines [18]. In addition, the prescription of fluoroquinolones, co-trimoxazole, and co-amoxiclav decreased significantly. The increase of fosfomycin prescription was in line with the alteration from third to second preference in 2013 of the national guideline. These data underscore the adherence of GPs to the national guidelines and indicate that national guidelines are valuable in antibiotic stewardship [45].

Although fosfomycin shows promising results of low resistance in UTI isolates [46] recently, a few studies describe less clinical efficacy [47, 48]. Based on the literature and the NHG guidelines, our recommendation is still nitrofurantoin as first choice, followed by fosfomycin and trimethoprim as third place if the isolated pathogen is susceptible. When more clinical studies become available concerning the (decreased) clinical efficacy of fosfomycin, the NHG guidelines might revise the recommendations.

## Strengths and limitations

The strength of our study is the national coverage, because the participating general practices are nationally representative for age, gender, regional distribution, and population density [21]. This enables us to compare (regional) differences in antibiotic resistance of uropathogenic *E. coli* over time and to evaluate whether the prescriptions of the GPs are in line with the recommendations of the NHG guideline for the treatment of uncomplicated UTI. The data we collected in our study is essential for the setup of guidelines for the treatment of uncomplicated UTI. Data in Nethmap are from patients after therapy failure and not representative for successfully treated patients and not suitable for the NHG guidelines [18].

A limitation of our study is the limited information regarding lifestyle, known risk factors, like co-morbidity, hospitalization, reinfection, or previous antibiotic use*.* More detailed information as to patient and environmental characteristics is recommended in the next survey. Also, our study is only representative for the group of healthy women. Men and children should be further investigated in a next survey to complete national guidelines for GPs in treatment of UTI.

In conclusion, the susceptibility percentages of *E. coli* to most antimicrobial agents isolated from uncomplicated UTI among women were stable in the Netherlands over 10 years’ time, although the prevalence of *E. coli* and ESBL-producing *E. coli* increased. These findings suggest that performing the survey at a regular interval is warranted. The GPs’ empirical prescription treatment was in line with the recommendations of the national NHG guidelines.
